# Machine learning-based predictive modeling of depression in hypertensive populations

**DOI:** 10.1371/journal.pone.0272330

**Published:** 2022-07-29

**Authors:** Chiyoung Lee, Heewon Kim

**Affiliations:** 1 School of Nursing & Health Studies, University of Washington Bothell, Bothell, Washington, United States of America; 2 The Department of Electrical and Computer Engineering, Automation and Systems Research Institute, Seoul National University, Seoul, Korea; Georgia State University, UNITED STATES

## Abstract

We aimed to develop prediction models for depression among U.S. adults with hypertension using various machine learning (ML) approaches. Moreover, we analyzed the mechanisms of the developed models. This cross-sectional study included 8,628 adults with hypertension (11.3% with depression) from the National Health and Nutrition Examination Survey (2011–2020). We selected several significant features using feature selection methods to build the models. Data imbalance was managed with random down-sampling. Six different ML classification methods implemented in the R package *caret*—artificial neural network, random forest, AdaBoost, stochastic gradient boosting, XGBoost, and support vector machine—were employed with 10-fold cross-validation for predictions. Model performance was assessed by examining the area under the receiver operating characteristic curve (AUC), accuracy, precision, sensitivity, specificity, and F1-score. For an interpretable algorithm, we used the variable importance evaluation function in *caret*. Of all classification models, artificial neural network trained with selected features (n = 30) achieved the highest AUC (0.813) and specificity (0.780) in predicting depression. Support vector machine predicted depression with the highest accuracy (0.771), precision (0.969), sensitivity (0.774), and F1-score (0.860). The most frequent and important features contributing to the models included the ratio of family income to poverty, triglyceride level, white blood cell count, age, sleep disorder status, the presence of arthritis, hemoglobin level, marital status, and education level. In conclusion, ML algorithms performed comparably in predicting depression among hypertensive populations. Furthermore, the developed models shed light on variables’ relative importance, paving the way for further clinical research.

## Introduction

Depression is a frequent comorbidity among individuals with hypertension. A meta-analysis including 41 studies demonstrated that 26.8% of patients with hypertension had depression [1]. Notably, depression is associated with inadequate blood pressure control and hypertension complications [2]. It also negatively affects patients’ adherence to treatments, health behavior, and quality of life, all of which may produce poorer long-term outcomes [3]. Given the significant burden depression poses on individuals with hypertension, its early prediction in this group is critical.

Today, machine learning (ML) has been helpful for researchers in designing optimal predictive models within and across large datasets. In the United States, ML has been applied to train a classification model that could accurately identify depression based on several demographic, social, and clinical factors, either in the general population [4, 5] or in people with chronic condition such as diabetes [4] or heart disease [6]. However, such efforts to identify depression in individuals with hypertension are lacking.

Accordingly, it is necessary to develop available ML models to screen for depression, facilitating early intervention in hypertensive populations. Model performance depends on both the research question and the type of data available. After appropriate data collection and processing (i.e., healthy data) and database building, evaluating models that are most suitable for the problem statement is crucial [7]. Several ML models crucial to clinical diagnosis of depression have been developed till date. Some techniques, such as artificial neural networks (ANNs), utilize deep learning algorithms and have successfully been applied in precision psychiatry [8]. In practice, ANN is a powerful non-linear statistical tool that can model complex associations between variables to best predict an outcome based on large-scale empirical data [9]. Other scholars have used conventional ML models, including ensemble methods such as random forests, AdaBoost, stochastic gradient boosting, and XGBoost, as well as advanced kernel-based techniques such as the support vector machine (SVM). All those models are useful in predicting psychiatric illness among patients with varying symptom severity, etiology, and clinical status [10].

In parallel, determining important factors contributing to depression is critical for early identification of individuals at risk of depression. In psychiatry, deep-learning algorithms are used to predict depression based on multimodal approaches such as using video, audio, and text streams. However, deep learning models based on textual and numeric data on clinical history and questionnaires tend to be non-explainable. Given that many ML methods, including ANN, are black boxes, they are limited in providing meaningful interpretations. Fortunately, nowadays several statistical packages offer an approach for researchers to interpret the models with visual presentations and clear interpretations of the analysis results [11].

### Aim of the study

The aim of this study was to develop ML-based predictive models for depression in individuals with hypertension. Particularly, we implemented the classification process using six different ML algorithms: ANN, random forest, AdaBoost, stochastic gradient boosting, XGBoost, and SVM. In addition, the variable importance evaluation function with the *caret* package in R software [12] was used to interpret the operating mechanisms of the ANN model and that of the conventional ML-based classification models. Using this function, the predictors were ranked according to their relative contribution to the variable importance for each model.

## Materials and methods

### Data source

We used the National Health and Nutrition Examination Survey (NHANES) datasets to train the model. NHANES is a periodic cross-sectional survey conducted by the Centers for Disease Control and Prevention to monitor trends in health and nutritional status in the non-institutionalized, community-dwelling US population. This survey has a complex multistage design to increase its representativeness. Approximately 5,000 individuals participate in the NHANES each year, and the data are reported in two-year cycles. Our study analyzed NHANES data from 2011–2020.

### Population

The study population included a national sample of adults (≥ 40 years) with hypertension. Consistent with previous research [13], hypertension was defined as meeting one of the following criteria: (a) ever been told to have high blood pressure; (b) ever been told to take prescription for hypertension; (c) now taking prescribed medicine for hypertension; or (d) having average systolic blood pressure greater than or equal to 140 mmHg or diastolic blood pressure greater than or equal to 90 mmHg in the NHANES examination section. During the NHANES 2011–2020 survey, 8,938 adults (aged ≥ 40 years) with hypertension were identified. Of those, 310 participants (3.5%) did not reply to the depression screening questionnaires (i.e., Patient Health Questionnaire-9 [PHQ-9]) and were hence excluded. This resulted in 8,628 participants eligible for inclusion.

### Ethical review

Ethical approval was not required as NHANES is a publicly available dataset that removed personal identifiers. In addition, the University of Washington institutional review board deemed this study as exempt.

### Measures

#### Inputs for predictive modeling

We selected factors potentially predicting depression based on data availability and previous studies’ findings [5, 14]. These included sociodemographic, behavioral, and clinical factors, as well as anthropometrics and biomarkers. In the NHANES, sociodemographic, behavioral, and clinical data are collected via home interview-administered questionnaires, while trained staff collect anthropometrics and biomarkers using mobile exam units.

Sociodemographic factors include age, race/ethnicity, gender, marital status, education level, the ratio of family income to poverty, insurance status, and time spent uninsured in the past year. Behavioral factors include smoking status, minutes of sedentary activity, vigorous work activity, moderate work activity, walking or cycling, vigorous recreational activity, and moderate recreational activity. Clinical factors include the presence of arthritis, kidney disease, asthma, liver disease, cancer or a malignance of any kind, cardiovascular disease, and sleep disorders. Participants were considered as prevalent cardiovascular disease cases if they had ever been told by a doctor that they had any of the following conditions: congestive heart failure, coronary heart disease, angina/angina pectoris, heart attack, or stroke. Sleep disorder was assessed with the question “Have you ever told a doctor or other health professional that you have trouble sleeping?” Anthropometric and biomarkers include segmented neutrophils number, white blood cell count, red cell distribution width, mean cell volume, platelet count, gamma glutamyl transferase, alanine aminotransferase, alkaline phosphatase, eosinophils number, basophils number, glycohemoglobin, triglycerides, total cholesterol, body mass index, direct high-density lipoprotein cholesterol, sodium, total bilirubin, hemoglobin, hematocrit, albumin, monocyte number, lymphocyte number, potassium, uric acid, and creatinine.

#### Outputs for predictive modeling: Depression

For the diagnosis of depression, we used the PHQ-9 [15]. The PHQ-9 consists of 9 items based on the diagnostic criteria for depression from the Diagnostic and Statistical Manual of Mental Disorders IV. Each item is rated on a 3-point scale basis the frequency of depressive symptoms (0 = “not at all” to 3 = “nearly every day”). Scores ranged from 0 to 27, with higher scores indicating a higher severity of depression. We selected 10 as our threshold for the diagnosis of depression, as this is a reliable threshold with acceptable sensitivity and specificity for detecting major depressive disorders [16]. During 2011–2020, 8,628 participants were assessed for depression and 976 received the diagnosis of depression (11.3%).

### Data analysis steps

All analyses were conducted in R version 4.1.1 and its packages. Most of the input variables used in the current study have a missing data rate of < 7.0%. Missing values were replaced by the mean for continuous data and by the mode for categorical data by using the “na.roughfix()” function in the *randomForest* package. Descriptive and bivariate analyses assessed baseline characteristics depending on participants’ depression status. Further, the ML findings were structured using the following steps: (1) feature selection, (2) data pre-processing and partitioning, (3) managing data imbalance, (4) ML analysis for predictive classification modeling, and (5) ranking variable importance.

#### Feature selection

Given that not all features carry significant information, feature selection to discard redundant features that can potentially deteriorate the model performance was conducted. We evaluated 3 data-driven feature selection methods—including (1) 2 ML algorithms (Boruta and the least absolute shrinkage and selection operator [LASSO]) and (2) stepwise backward elimination—and selected whichever one could produce the best informative feature sets for our final prediction.

Boruta is a wrapper algorithm built around random forests that finds all relevant attributes by comparing the importance of the randomized copies of the attributes with that of the original attributes [17]. LASSO is a regression method, which performs variable selection and regularization using L1 penalty to shrink regression coefficients of the redundant features to zero [18]. In the current study, the penalty parameter lambda (λ) was tuned using 10-fold cross-validation based on the minimum partial likelihood deviance. The features with nonzero coefficients in optimal λ were selected and used in the model. Backward elimination removes predictor variables insignificant to the model based on the Akaike information criterion value, until the ideal number of predictor variables is achieved [19]. We used the *Boruta* package for Boruta, *glmnet* package for LASSO, and *MASS* package for stepwise backward elimination.

#### Data pre-processing and partitioning

To transform the raw data into the appropriate format for the ML model building, the datasets with the finalized features were preprocessed using the scale and center transformation methods of the “preProcess()” function in the *caret* package. Furthermore, all the categorical variables were one-hot encoded and were encoded as 2-factor variables. After the data were pre-processed, they were randomly divided into 2 sets: training (80.0%) and testing (20.0%). The training dataset was used to “train” and finalize the optimal model, whereas the testing dataset was used to evaluate the performance of the final model.

#### Managing data imbalance

The dataset used had extreme class imbalance (depression prevalence of 11.3%). ML algorithms tend to be biased toward the majority class and always return higher accuracy, which can be misleading. We used the random down-sampling technique provided by the *ROSE* package [20] to handle this imbalance. Random down-sampling was chosen as it performed better than random oversampling or the synthetic minority over-sampling technique (SMOTE) [21] in our datasets, despite its simplicity. After down-sampling, the sizes of the 2 classes in the training data were similar (745, non-depressed: 781, depressed each).

#### ML analysis for predictive classification modeling

We performed classification modeling to predict the binary class of depression using features returned by feature selection methods. The modeling function in the *caret* package was used for all predictions to ensure uniform execution: ‘nnet’ (ANN), ‘rf’ (random forest), ‘adaboost’(AdaBoost), ‘gbm’(stochastic gradient boosting), ‘xgbtree’ (XGBoost), and ‘svmLinear’ (SVM) (see Table 1 for further details on each specific method). To identify and decrease the error values during model fitting, determining the optimal hyperparameters for each of the ML algorithms is crucial. Hyperparameter tuning is streamlined and easy to use in *caret* [12]. By default, the *caret* package automatically tunes the hyperparameter values for each algorithm using the package’s standard grid set of candidate models. We then applied these hyperparameters to the down-sampled training data to fit the model parameters. Model parameters then tested the data to evaluate model performance. Cross-validation was used to select the best set of parameters for the final prediction; all models were trained with 10-fold cross-validation with 3 replications.

**Table 1:** ML algorithms used in the current study [24].

	Description	Hyperparameters used in *caret*
**ANN**	ANN is a group of interconnected artificial neurons that utilizes a mathematical model or computational model to process information. The generic structure of a basic ANN comprises a series of nodes arranged in 3 layers (input, hidden, and output layers). The input nodes and the output node of an ANN correspond to the predictor variables and outcome variable, respectively. The nodes in the hidden layer are intermediate unobserved values that allow the ANN to model complex nonlinear associations between the input nodes and the output node. The nodes in different layers are connected by weights.	hidden layer = 1, decay weight = 0.09
**Random forest**	Random forest is a tree-based ensemble method that utilizes parallel decision trees built on subsets of the data to develop an optimal predictive model. Each tree in the random forest casts a vote based on its prediction, and the classification with the most votes becomes the overall model’s prediction.	mtry = 2
**AdaBoost**	AdaBoost is also an ensemble method like random forest. The core principle of AdaBoost is to fit a sequence of “weak learners” (i.e., models that are only slightly better than random guessing) to repeatedly modified data. All predictions are then combined through a weighted majority vote (or sum) to generate the final prediction.	nIter = 100, method = Adaboost.M1
**Stochastic gradient boosting**	Stochastic gradient boosting is another ensemble technique. It iteratively builds several small decision trees, each based on a random subset of the data, with each additional tree emphasizing observations poorly modeled by the existing collection of trees. Ultimately, observations are assigned a class based on the most common classification among the trees.	n.trees = 100, interaction.depth = 1, shrinkage = 0.1, and n.minobsinnode = 10
**XGBoost**	XGBoost implements gradient boosting with decision trees as the underlying learners. Whereas random forest employs individual trees in parallel to solve the same problem, XGBoost builds individual trees sequentially. Each tree is trained to resolve the prediction error remaining following the prior tree and thereby improves prediction. This offers another approach to building more complex and accurate models with trees while controlling individual tree depth and complexity.	nrounds = 1000, max_depth = 10, eta = 0.07, gamma = 0.01, colsample_bytree = 0.5, min_child_weight = 1, and subsample = 0.5
**SVM** ^a^	An SVM model represents data samples as points in a space. The samples of separate categories are divided by a clear gap that should be as wide as possible. New data samples are then mapped onto that same space and predicted to become part of a category based on the side of the gap onto which they are mapped.	C = 0.1

^a^In this study, we chose the linear kernel function as the kernel function of the SVM classifier

ANN: artificial neural network; SVM: support vector machine

Model performance was assessed by examining the area under the receiver operating characteristic curve (AUC), accuracy, precision, sensitivity, specificity, and F1-score. AUC is a widely used metric for binary classification problems and provides a representative summary of the performance of a classifier. Generally, AUC values of 0.8 to 0.9 are considered good and above 0.9 are considered excellent [22]. A higher F1-score signifies less false-positives and less false-negatives, which implies correct identification of the classes [22]. Both AUC and F1-score are well-known metrics for classification performance evaluation over an imbalanced dataset [23]. Accuracy, precision, sensitivity, specificity, and F1-score were evaluated using a confusion matrix. S1 Table details the calculation methods for these diagnostic performance measures.

#### Ranking variable importance

We used the ‘varImp()’ function of the *caret* package to determine the relative predictor importance for each model. Using this function, the predictors were ranked according to their relative contribution to the variable importance for each model.

## Results

The results were “unweighted” as we could not accommodate a complex survey design into the analyses due to the current lack of ML methodologies for handling complex design features (e.g., sampling weights, strata, and primary sampling units). Hereafter, we refer to unweighted prevalence rates (or unweighted means) directly as prevalence rates (or means) and provide further discussion in the limitations section.

### Comparison of baseline characteristics

Among the 8,628 adults in the sample with hypertension, 976 (11.3%) reported a clinical level of depression based on their PHQ-9 score. The depressed group was significantly younger than the nondepressed group. This group had a higher percentage of individuals with Mexican, other Hispanic, and “other” ancestry, whereas the nondepressed group had a higher percentage of non-Hispanic White, non-Hispanic Black, and non-Hispanic Asian individuals. Women comprised 63.8% of the depressed group. More than half of those in the nondepressed group were married or living with a partner and had a college degree or more education. The ratio of family income to poverty in the nondepressed group was significantly higher than that in the depressed group. Table 2 provides additional characteristics of participants subdivided by depression status.

**Table 2:** Comparison of baseline characteristics (unweighted).

Variables	n (%) or mean ± SD		*t* or *χ*^*2*^	*p*-value
	Non-depressed (n = 7,652)	Depressed (n = 976)		
**Sociodemographic factors**				
Age	63.31 ± 11.40	61.32 ± 10.68	5.43	< 0.001
Race/ethnicity			56.69	< 0.001
Mexican American	740 (9.7)	121 (12.4)		
Other Hispanic	748 (9.8)	126 (12.9)		
Non-Hispanic White	2,989 (39.1)	376 (38.5)		
Non-Hispanic Black	2,282 (29.8)	268 (27.5)		
Non-Hispanic Asian	665 (8.7)	35 (3.6)		
Other	228 (3.0)	50 (5.1)		
Gender			70.99	< 0.001
Male	3,863 (50.5)	353 (36.2)		
Female	3,789 (49.5)	623 (63.8)		
Marital status			102.17	< 0.001
Married/living with partner	4,449 (58.2)	401 (41.1)		
Widowed/divorced/separated	2,502 (32.7)	449 (46.1)		
Never married	696 (9.1)	125 (12.8)		
Education level			136.09	< 0.001
Less than 9^th^ grade	754 (9.8)	165 (16.9)		
9-11^th^ grade	984 (12.9)	192 (19.7)		
High school graduate/GED or equivalent	1,914 (25.0)	242 (24.8)		
Some college or AA degree	2,335 (30.5)	286 (29.3)		
College graduate or above	1,665 (21.8)	91 (9.3)		
The ratio of family income to poverty^a^	2.58 ± 1.60	1.65 ± 1.30	19.49	< 0.001
Insurance status			3.04	0.080
Yes	6,828 (89.3)	851 (87.5)		
No	817 (10.7)	122 (12.5)		
Time spent uninsured in the past year			15.42	< 0.001
Yes	309 (4.5)	65 (7.6)		
No	6,547 (95.5)	795 (92.4)		
**Behavioral factors**				
Smoking status			30.48	< 0.001
Yes	3,713 (48.6)	565 (58.0)		
No	3,933 (51.4)	410 (42.0)		
Minutes of sedentary activity	373 ± 200.6	404.4 ± 224.3	-4.13	< 0.001
Vigorous work activity			2.79	0.090
Yes	1,294 (16.9)	186 (19.1)		
No	6,355 (83.1)	790 (80.9)		
Moderate work activity			0.63	0.430
Yes	2,621 (34.3)	322 (33.0)		
No	5,026 (65.7)	654 (67.0)		
Walking or cycling			0.44	0.510
Yes	1,535 (20.1)	187 (19.2)		
No	6,115 (79.9)	789 (80.8)		
Vigorous recreational activity			51.32	< 0.001
Yes	958 (12.5)	46 (4.7)		
No	6,693 (87.5)	930 (95.3)		
Moderate recreational activity			92.68	< 0.001
Yes	2,877 (37.6)	214 (21.9)		
No	4,771 (62.4)	762 (78.1)		
**Clinical factors**				
Presence of arthritis			185.50	< 0.001
Yes	3,375 (44.2)	654 (67.4)		
No	4,261 (55.8)	317 (32.6)		
Presence of kidney disease			51.01	< 0.001
Yes	512 (6.7)	127 (13.1)		
No	7,128 (93.3)	844 (86.9)		
Presence of asthma			100.46	< 0.001
Yes	1,138 (14.9)	268 (27.5)		
No	6,512 (85.1)	708 (72.5)		
Presence of liver disease			67.15	< 0.001
Yes	468 (6.1)	128 (13.2)		
No	7,174 (93.9)	842 (86.8)		
Presence of cancer or a malignance of any kind			1.24	0.265
Yes	1,251 (16.4)	173 (17.8)		
No	6,398 (83.6)	801 (82.2)		
Presence of cardiovascular disease^b^			77.94	< 0.001
Yes	1,541 (20.4)	311 (33.0)		
No	6,007 (79.6)	631 (67.0)		
Presence of sleep disorder			478.04	< 0.001
Yes	2,461 (32.2)	662 (67.9)		< 0.001
No	5,190 (67.8)	313 (32.1)		
**Anthropometric and biomarkers**				
Segmented neutrophils number (1000c cells/uL)	4.27 ± 1.70	4.66 ± 2.04	-5.57	< 0.001
White blood cell count (1000 cells/uL)	7.28 ± 5.25	7.72 ± 2.53	-4.25	< 0.001
Red cell distribution width (%)	13.93 ± 1.42	14.17 ± 1.54	-4.52	< 0.001
Mean cell volume (fL)	89.48 ± 6.15	89.21 ± 6.36	1.27	0.205
Platelet count (1000 cells/uL)	233.1 ± 64.22	245.3 ± 71.26	-4.96	< 0.001
Gamma glutamyl transferase (U/L)	33.91 ± 50.52	31.91 ± 61.31	-3.75	< 0.001
Alanine aminotransferase (U/L)	23.49 ± 18.39	25.71 ± 47.56	-1.39	0.008
Alkaline phosphatase (U/L)	75.48 ± 27.92	81.01 ± 27.93	-5.60	< 0.001
Eosinophils number (1000 cells/uL)	0.21 ± 0.18	0.22 ± 0.17	-1.43	0.154
Basophils number (1000 cells/uL)	0.05 ± 0.05	0.06 ± 0.05	-2.60	0.009
Glycohemoglobin (%)	6.15 ± 1.22	6.40 ± 1.58	-4.55	< 0.001
Triglyceride (mmol/L)	1.79 ± 1.29	2.01 ± 1.50	-4.26	< 0.001
Total cholesterol (mmol/L)	4.89 ± 1.13	4.98 ± 1.18	-2.11	0.035
Body mass index (kg/m^2^)	30.71 ± 7.15	32.81 ± 8.53	-7.26	< 0.001
Direct high-density lipoprotein cholesterol (mmol/L)	1.38 ± 0.43	1.35 ± 0.46	1.68	0.094
Sodium (mmol/L)	139.7 ± 2.76	139.7 ± 2.94	0.44	0.661
Total bilirubin (umol/L)	9.69 ± 4.84	8.86 ± 4.47	5.17	< 0.001
Hemoglobin (g/dL)	13.82 ± 1.55	13.54 ± 1.65	4.93	< 0.001
Hematocrit (%)	41.16 ± 4.30	40.45 ± 4.60	4.46	< 0.001
Albumin, urine (mg/L)	84.52 ± 438.8	130.2 ± 534.5	-2.53	0.010
Albumin, refrigerated serum (g/L)	41.41 ± 3.39	40.53 ± 3.73	6.73	< 0.001
Monocyte number (1000 cells/uL)	0.59 ± 0.24	0.60 ± 0.22	-1.88	0.060
Lymphocyte number (1000 cells/uL)	2.16 ± 4.48	2.18 ± 0.82	-0.29	0.772
Potassium (mmol/L)	4.04 ± 0.41	4.04 ± 0.43	-0.38	0.703
Uric acid (umol/L)	343.7 ± 89.79	337.5 ± 94.54	1.93	0.054
Creatinine, urine (umol/L)	10290.7 ± 6677.7	10771.2 ± 7273.8	-1.93	0.054

^a^The variable of family annual income was computed as a ratio of family income to poverty guidelines using the federal poverty level guidelines, which were available at (https://aspe.hhs.gov/prior-hhs-poverty-guidelines-and-federal-registerreferences). The poverty index is a ratio measuring the household income to the poverty threshold after accounting for inflation and family size.

^b^Participants were considered as prevalent cardiovascular disease cases if ever told by a doctor that they had any of the following conditions: congestive heart failure, coronary heart disease, angina/angina pectoris, heart attack, or stroke.

SD: standard deviation

### Feature selection for modeling

Among the three different feature selection techniques, stepwise backward elimination showed the most substantial reduction in the number of features (from 47 to 30; see S2 Table and S1 Fig) and yielded the optimal predictive performance (see S3 Table). Thus, we primarily based our models on features selected from the stepwise backward elimination method (see Supporting information files for full feature selection results). Features selected by stepwise backward elimination included: age, race/ethnicity, gender, marital status, education level, the ratio of family income to poverty, time spent uninsured in the past year, smoking status, minutes of sedentary activity, vigorous work activity, vigorous recreational activity, moderate recreational activity, all clinical factors, white blood cell count, platelet count, alanine aminotransferase, glycohemoglobin, triglycerides, total cholesterol, sodium, hemoglobin, lymphocyte number, uric acid, and creatinine.

### ML analysis for predictive modeling

In the current study, the ANN model trained with selected features (n = 30) was developed with 1 hidden layer, and the decay weight was set at 0.09 based on cross-validation as it yielded the highest test set accuracy. ANNs were also tested using the *keras* package with varying depth, size of hidden layers, and regularization (dropout and L2 penalty); however, no combination of hyperparameters tested yielded a higher AUC than the *caret* implementation. Table 1 summarizes the hyperparameters used in other models.

**Fig 1:**
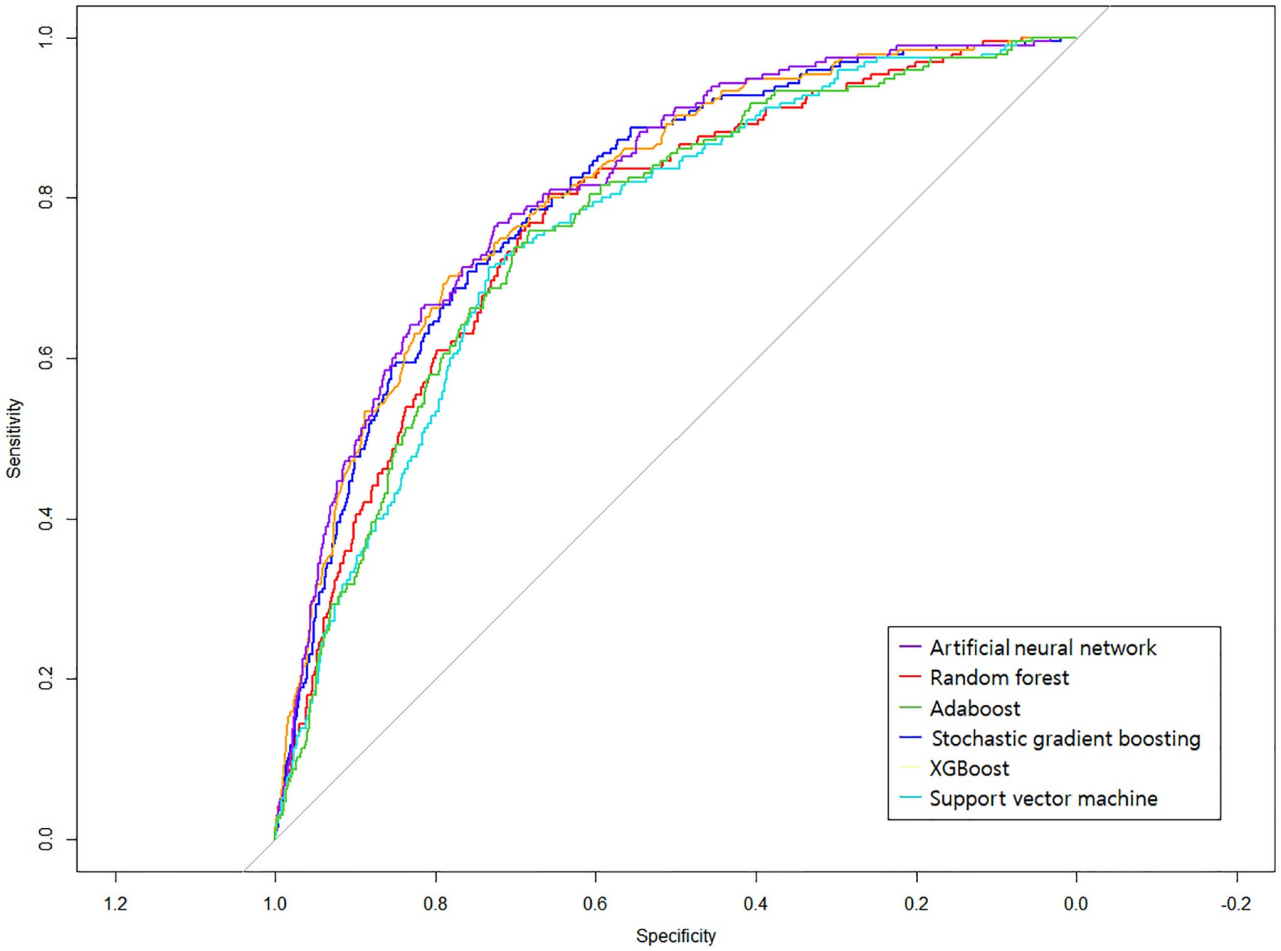
ROC curves for six machine learning models in predicting depression. Ten-fold cross-validation was used to build and evaluate the prediction models. Different colors represent the different machine learning classifiers used in this study. The gray line is the reference corresponding to the performance of a classifier that completely and randomly classifies the condition.

The six ML models’ classification performance based on the features selected from stepwise backward elimination is illustrated in an ROC curve (Fig 1). Of all classification models, ANN achieved the highest AUC (0.813) and specificity (0.780) in predicting depression. SVM predicted depression with the highest accuracy (0.771), precision (0.969), sensitivity (0.774), and F1-score (0.860). All classifiers achieved better classification accuracy than a random model (the gray diagonal line indicating AUC = 0.500 in Fig 1). Table 3 further demonstrates other model’s performance.

**Table 3:** Average metrics of six models trained with stepwise backward elimination.

Model	AUC	Accuracy	Precision	Sensitivity	Specificity	F1-score
**ANN**	**0.813**	0.706	0.961	0.697	**0.780**	0.808
**Random forest**	0.772	0.686	0.958	0.676	0.769	0.792
**AdaBoost**	0.762	0.673	0.956	0.662	0.759	0.782
**Stochastic gradient boosting**	0.803	0.707	0.956	0.701	0.748	0.809
**XGBoost**	0.808	0.696	0.958	0.688	0.764	0.801
**SVM**	0.760	**0.771**	**0.969**	**0.774**	0.739	**0.860**

The highest value was bolded.

AUC: area under the receiver operating characteristic curve; ANN: artificial neural network; SVM: support vector machine

### Important features ranked by ML algorithm

The selected features contributed differently to each model. We combined the top 20 strongest contributing features from the six models and ranked them based on their inclusion in the models. In total, these models returned 24 top-20 features, nine of which were within the top 20 in at least five models, based on their rankings in each model (Table 4). The most frequent and important features include: the ratio of family income to poverty, triglyceride level, white blood cell count, age, sleep disorder status, the presence of arthritis, hemoglobin level, marital status, and education level.

**Table 4:** The most contributing features belonging to at least five of the six models.

Features	Frequency	Rank						Descriptions
		ANN	Random forest	AdaBoost	Stochastic gradient boosting	XGBoost	SVM	
**The ratio of family income to poverty**	6	1	1	2	1	1	2	The ratio of family income to poverty guidelines
**Triglycerides**	6	14	4	10	4	3	10	Triglycerides, refrigerated serum (mmol/L)
**White blood cell count**	6	13	7	13	11	5	13	White blood cell count (1000 cells/uL)
**Age**	6	9	9	11	14	8	11	Age in years of the participant at the time of screening
**Sleep disorder**	5	2	2	1	2	NA	1	Ever told a doctor that you had trouble sleeping
**Arthritis**	5	8	16	3	3	NA	3	Ever told by a doctor that you had arthritis
**Hemoglobin**	5	NA	6	12	16	6	12	Hemoglobin (g/dL)
**Marital status**	5	NA	18	5	10	16	5	Marital status of the participants
**Education level**	5	15	17	8	8	NA	8	The highest grade or level of schooling or the highest degree

ANN: artificial neural network; SVM: support vector machine

NA: This feature was not ranked in the top 20 features for that model.

## Discussion

The ML models developed in this study showed comparable performance in predicting depression among U.S. adults with hypertension. ANN specifically achieved the highest performance in terms of AUC and specificity. Few studies that have evaluated ANN-based models for predicting psychiatric illnesses have consistently outperformed conventional ML methods and traditional regression models [5, 25, 26]. Our findings add to the evidence of ANN models’ power as computational tools for early diagnosis of depression in individuals with chronic conditions. Nevertheless, although not directly comparable, our ANN model’s predictive ability was comparably lower than that found in previous ML studies, in which AUC values ranged from 0.910 to 0.920 [5] or equal to 0.913 [27].

Notably, SVM also exhibited strong predictive performance with respect to other diagnostic measures, including accuracy, precision, sensitivity, and F1-score. SVM has recently gained crucial importance as neural network approaches for predicting the diagnosis and prognosis of a range of psychiatric and neurological disorders, including Alzheimer’s disease, schizophrenia, and depression [28–30]. Of note, the SVM has a high predictive accuracy when using large biomedical datasets comprising a small number of records with a large number of variables (i.e., insensitivity to high-dimensional data) and is less affected by imbalanced datasets [23, 31], making it suitable for our analysis. However, SVMs do not always show a high predictive accuracy; in several papers, RF-based models have been reported to perform equally well or better than other algorithms [32–36]. For instance, in the studies by Mousavian et al. [33] and de Souza Filho et al. [34], RF outperformed SVM in predicting depression. Similarly, RF had the best accuracy in predicting anxiety, depression, and stress in the study by Priya et al. [35].

The ratio of family income to poverty was the most important feature across all models. This result accords with findings of recent ML studies [4, 37], which reported the ratio of family income to poverty (or family income itself) as the most crucial feature in predicting depression among community-dwelling adults. Kang and Kim [38] also have noted that the associations of hypertension with symptoms and diagnosis of depression differ by income level. In addition to income, factors such as age, marital status, education—all of which are “social determinants of mental health,” per Carod-Artal [39]—were also among the most important features across the models. Age has consistently been identified as a critical factor in explaining the variability in depression prevalence rates [40]. Marital status is one of the most important social factors affecting various life outcomes, especially mental health [39]. Education strongly affects depression as it heightens cognitive ability, provides economic and social resources, and leads to positive health behaviors [41]. Based on our results, we recommend that, in addition to the usual variables, healthcare providers collect information regarding these social determinants at the earliest possible opportunity to prevent depression and to screen individuals with hypertension for depression risk.

We identified several important biomarkers across the model: triglycerides, white blood cell count, and hemoglobin. In Lin et al.’s study [4] using random forest, triglycerides were an essential variable in building a depression prediction model that included the general population and individuals with a high body mass index. Sharma and Verbeke [14] observed that triglycerides were an important biomarker for diagnosing and distinguishing depression cases from healthy cases using the XGBoost algorithm. Moreover, non-ML studies demonstrate that higher depression scores are associated with an enhanced inflammatory state, as evidenced by higher levels of hematological inflammatory markers including white blood cells, both in individuals free of disease [42] and those with stable heart disease [43]. Finally, among less known modifiable risk factors for depression, anemia has attracted increasing attention. Anemia is often associated with conditions (e.g., cancer, chronic renal failure, malnutrition, etc.) that usually precede depressed mood [44]. Symptoms of low hemoglobin levels (e.g., paleness, fatigue, dizziness, shortness of breath during physical activity, etc.) also frequently occur alongside depressive symptoms [45]. Some have proposed that anemia has a pathophysiological role in depression due to chronic hypo-oxygenation [46, 47], which further supports the importance of including hemoglobin levels in our model.

Sleep disorder status was also important in building the predictive model. Disturbed sleep is associated with metabolic, neuroendocrine, and inflammatory changes, resulting in alterations to mental functioning [48]. Ma and Li [49] have also reported a significant correlation between sleep quality and depression in older patients with hypertension. Of note, assessment of sleep disorders in individuals with hypertension pertain is important not only for preventing comorbid debilitating mental health disorders but also for mitigating their adverse influence on hypertension management. According to one hypotheses, sleep alterations may impair adaptation to stress through allostasis and contribute to allostatic load, thereby compromising stress resiliency and amplifying blood pressure [50]. Faraut et al. [51] found that participants with short and long-sleep durations were more likely to have depressive symptoms, higher social vulnerability, and higher hypertension rates. One limitation in our interpretation is that the type of sleep disorder was not reported in the NHANES survey. Therefore, we could not explore the specific association between different sleep disorders and depression in the population with hypertension, which should be addressed in future studies. Such information may have helped derive more precise insights in preventing depression in our target population.

Lastly, arthritis was among our models’ most important features. It has long been recognized that arthritis and depression are associated [52]. Individuals with arthritis fear long-term pain, loss of function, work disability, and possible socioeconomic effects of the disease [53]. With these rational fears and physical challenges, most patients with arthritis exhibit clinically significant levels of low self-esteem and self-stigma, which explains the high prevalence of depressive disorders among these patients [54, 55]. Of importance, depression and arthritis increase the burden on the healthcare system, with increased provider visits, more pain complaints, and increased requests for pain medication that complicates hypertension management [56]. Therefore, greater primary preventive effort for depression should be directed toward individuals with hypertension and arthritis; for instance, administering routine depression screening.

### Limitations and implications

Our study has several limitations. First, although our ANN and SVM models evidently provided the best performance on the test set with the highest AUC and F1-score, the clinical utility of the models remains speculative at this stage. This is mainly because our results were based on self-reported data: the diagnosis of depression was based on a self-reported questionnaire in the present study without validation using actual clinical records or direct patient examination. In addition, our sample size was relatively small compared to other population studies. By building a more extensive database for training a prediction model, the variations observed among adults with depression can be more thoroughly incorporated. In the future, this may result in models with true clinical utility. Second, associations between inputs and outputs for predictive modeling do not infer causal relationships as the current study used cross-sectional data; for instance, the relationship between arthritis and depression may have been bidirectional. Another limitation is the inability of most ML algorithms to account for complex survey designs with multi-stage stratified sampling, which is often used for household surveys like the NHANES. Therefore, our sample should not be considered a true representative of community-dwelling adults with depression between 2011–2020.

Finally, recognizing the limitations of prevalence analyses is important. For instance, in the current study, the estimates of hypertension prevalence were drawn from many sources including survey data. However, an isolated survey response does not guarantee the diagnosis of the disease. In addition, antihypertensive medications can be prescribed to patients without hypertension; for example, the use of angiotensin-converting enzyme inhibitors for diabetic patients with chronic kidney disease. Accordingly, some samples included in the study may not have been representative of the population we targeted. Furthermore, the results should be interpreted cautiously since prevalence data alone cannot completely explain the disease dynamics. For example, in the case of a sleep disorder, a participant could have returned to sleep normalcy after medical treatment. Therefore, the question about the prevalence of sleep disorder does not assess whether the patient continued to have a sleeping disorder. Despite its limitations, this study is the first to predict depression among hypertensive populations using multiple ML approaches. This study also presents a potential method to aid the preliminary screening of depression among patients with hypertension, before a formal clinical diagnosis.

Several implications should be considered. First, our use of cross-sectional data to evaluate the ML models may have introduced bias in performance estimation, as the ML models’ performance should ideally be evaluated on newly collected data or a separate dataset for reliability. Further studies should address this limitation. Second, apart from including traditional risk factors, including different types of inputs could help further improve depression prediction [34]. For example, we did not include quality of life variables, such as familial relationships, social relationships, or leisure activity, which can help in better predicting depression prediction [37], owing to the fundamental limitations of the original NHANES survey. Third, the models developed in this study determined the variables’ predictive importance, facilitating additional clinical research; for instance, the strongest features across the models could be used to further improve depression prediction in future studies. Finally, a larger volume of data from the healthy population would be preferrable. With larger datasets, the methods employed will begin to vary and demonstrate improved validity [10]: particularly, the feature selection methods will improve performance, as they are likely to be affected by sample size; in addition, the k-fold cross-validation method can be utilized with larger k-values instead of the leave-one-out method to allow for larger sets on which to test prediction models and improve models’ generalizability.

## Conclusion

In the current study, ML algorithms performed comparably in predicting depression among U.S. adults with hypertension. Models with superior performance may aid in developing screening tools for depression among hypertensive adults in future studies. Furthermore, the risk factors for depression identified across the models may inform healthcare professionals to devise effective prevention strategies by focusing on at-risk individuals and may assist patients with hypertension with decisions regarding the use of diagnostic testing, treatments, or lifestyle changes.

## Supporting information

S1 Fig Feature selection figures (Boruta result plot), diagnostic plot of the Boruta algorithm (line plot of Z-scores), and cross-validated deviance with the number of non-zero coefficients and Lambda fit by LASSO logistic regression. (DOCX)

S1 Table The 2 X 2 contingency table. (DOCX)

S2 Table The list of variables selected from the Boruta and LASSO algorithms and stepwise backward elimination. (DOCX)

S3 Table Average metrics of six models trained with Boruta and LASSO algorithms. (DOCX)

S4 Table Codebook. (DOCX)
