# Supplementary material for: Machine learning-based predictive modeling of depression in hypertensive populations
Source: PLoS One. 2022 Jul 29;17(7):e0272330. doi: 10.1371/journal.pone.0272330 (PMC9337649; doi:10.1371/journal.pone.0272330)
Supplement: S1 Fig — (DOCX) [file pone.0272330.s001.docx]

# **S1A Fig. Feature selection figures (Boruta result plot).**


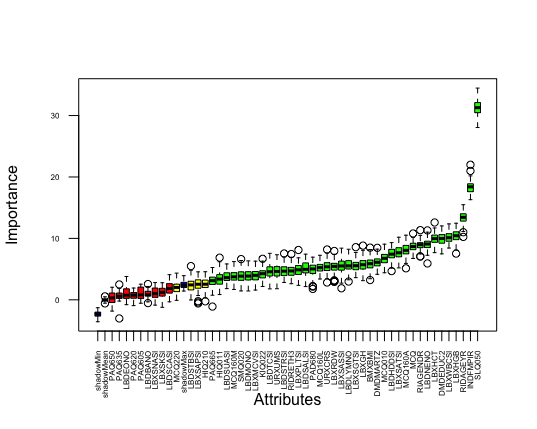


Blue boxplots correspond to minimal, average, and maximum Z score of a shadow attribute. Red boxplots represent rejected attributes, yellow boxplots represent tentative attributes, and green boxplots represent confirmed attributes.

# **S1B Fig. Diagnostic plot of the Boruta algorithm (line plot of Z-scores).**


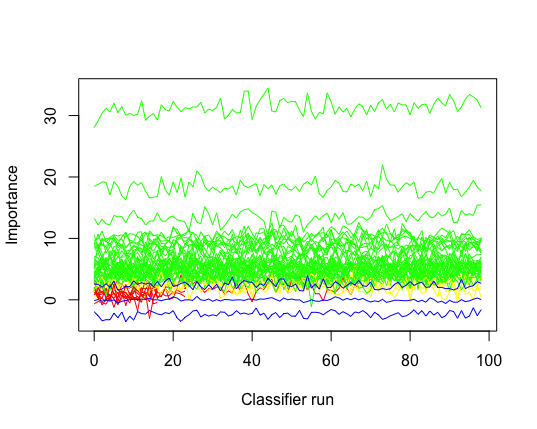


Green lines correspond to confirmed attributes, red to rejected ones, yellow to tentative ones, and blue to respectively minimal, average, and maximal shadow attribute importance. Gray lines separate rounds.

# **S1C Fig. Cross-validated deviance with the number of non-zero coefficients and Lambda fit by LASSO logistic regression.**


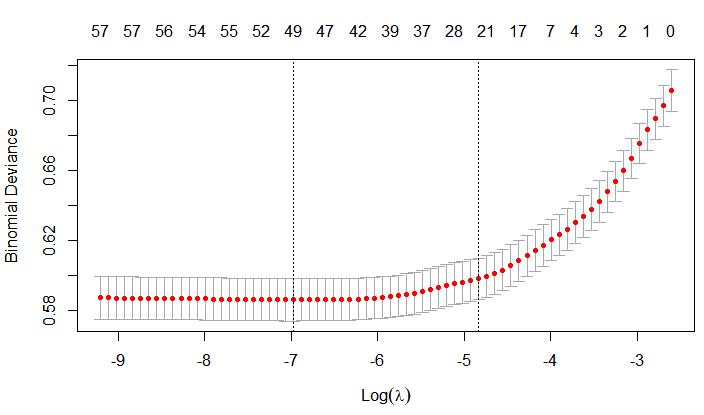


S3 Fig describe the mapping relationship of the cross-validated deviance with non-zero coefficients (that is, the number of selected radiomics features) and the lambda fit by LASSO logistic regression. The top axis is the number of non-zero coefficients. The left vertical dashed line represents the λ providing the smallest cross-validated deviance. In addition, the right vertical dashed line represents the minimum deviance plus no more than one standard deviation.

# **S3 Table. Average metrics of six models trained with Boruta and LASSO algorithms.**

| **Boruta** | | | | | | |
| --- | --- | --- | --- | --- | --- | --- |
| **Model** | **AUC** | **Accuracy** | **Precision** | **Sensitivity** | **Specificity** | **F1-score** |
| **ANN** | **0.803** | 0.698 | 0.958 | 0.692 | **0.771** | 0.803 |
| **Random forest** | 0.771 | 0.685 | 0.948 | 0.675 | 0.759 | 0.790 |
| **AdaBoost** | 0.760 | 0.670 | 0.952 | 0.660 | 0.749 | 0.779 |
| **Stochastic gradient boosting** | 0.799 | 0.700 | 0.949 | 0.699 | 0.743 | 0.801 |
| **XGBoost** | 0.801 | 0.706 | 0.956 | 0.683 | 0.762 | 0.800 |
| **SVM** | 0.759 | **0.761** | **0.966** | **0.729** | 0.721 | **0.843** |
| **LASSO** | | | | | | |
| **ANN** | **0.811** | 0.703 | 0.955 | 0.646 | 0.760 | 0.800 |
| **Random forest** | 0.770 | 0.684 | 0.953 | 0.670 | **0.763** | 0.789 |
| **AdaBoost** | 0.760 | 0.669 | 0.946 | 0.658 | 0.733 | 0.778 |
| **Stochastic gradient boosting** | 0.800 | 0.700 | 0.950 | 0.699 | 0.739 | 0.808 |
| **XGBoost** | 0.803 | 0.690 | 0.954 | 0.681 | 0.760 | 0.789 |
| **SVM** | 0.763 | **0.769** | **0.960** | **0.765** | 0.729 | **0.851** |

The highest value was bolded.

AUC: area under the receiver operating characteristic curve; ANN: artificial neural network; SVM: support vector machine

# **S4 Table. Codebook.**

|  | **Code (year)** | **Variable** | **Question/Description** | **Answer categories** |
| --- | --- | --- | --- | --- |
| **Target variable for predictive modeling** | DPQ010 (2011-2020) | Have little interest in doing things | [Over the last 2 weeks, how often have you been bothered by the following problems:] little interest or pleasure in doing things? | 0. Not at all  1. Several days  2. More than half the days  3. Nearly every day  7. Refused  9. Don’t know  (.) Missing |
|  | DPQ020 (2011-2020) | Feeling down, depressed, or hopeless | [Over the last 2 weeks, how often have you been bothered by the following problems:] feeling down, depressed, or hopeless? |  |
|  | DPQ030 (2011-2020) | Trouble sleeping or sleeping too much | [Over the last 2 weeks, how often have you been bothered by the following problems:] trouble falling or staying asleep, or sleeping too much? |  |
|  | DPQ040 (2011-2020) | Feeling tired or having little energy | [Over the last 2 weeks, how often have you been bothered by the following problems:] feeling tired or having little energy? |  |
|  | DPQ050 (2011-2020) | Poor appetite or overeating | [Over the last 2 weeks, how often have you been bothered by the following problems:] poor appetite or overeating? |  |
|  | DPQ060 (2011-2020) | Feeling bad about yourself | [Over the last 2 weeks, how often have you been bothered by the following problems:] feeling bad about yourself - or that you are a failure or have let yourself or your family down? |  |
|  | DPQ070 (2011-2020) | Trouble concentrating on things | [Over the last 2 weeks, how often have you been bothered by the following problems:] trouble concentrating on things, such as reading the newspaper or watching TV? |  |
|  | DPQ080 (2011-2020) | Moving or speaking slowly or too fast | [Over the last 2 weeks, how often have you been bothered by the following problems:] moving or speaking so slowly that other people could have noticed? Or the opposite - being so fidgety or restless that you have been moving around a lot more than usual? |  |
|  | DPQ090 (2011-2020) | Thoughts you would be better off dead | [Over the last 2 weeks, how often have you been bothered by the following problems:] Thoughts that you would be better off dead or of hurting yourself in some way? |  |
| **Predictors** | **Sociodemographic factors** | | | |
|  | RIDAGEYR (2011-2020) | Age in years at screening | Age in years of the participant at the time of screening. Individuals 80 and over are topcoded at 80 years of age. | Continuous  (.) Missing |
|  | RIDRETH3 (2011-2020) | Race/ethnicity | Recode of reported race and Hispanic origin information, with Non-Hispanic Asian Category | 1. Mexican American  2. Other Hispanic  3. Non-Hispanic White  4. Non-Hispanic Black  5. Non-Hispanic Asian  7. Other Race- Including Multi-Racial  (.) Missing |
|  | RIAGENDR (2011-2020) | Gender | Gender of the participant | 1. Male  2. Female  (.) Missing |
|  | DMDMARTZ (2017-2020)  DMDMARTL (2011-2016) | Marital status | Marital status | [2017-2020]  1. Married/living with Partner  2. Widowed/divorced/separated  3. Never married  77. Refused  99. Don’t know  (.) Missing  [2011-2016]  1. Married  2. Widowed  3. Divorced  4. Separated  5. Never married  6. Living with partner  77. Refused  99. Don’t know  (.) Missing |
|  | DMDEDUC2 (2011-2020) | Education level | What is the highest grade or level of school {you have/SP has} completed or the highest degree {you have/s/he has} received? | 1. Less than 9th 2. grade  3. 9-11th grade (Includes 12th grade with no diploma)  4. High school graduate/GED or equivalent  5. Some college or AA degree  6. College graduate or above  7. Refused  9. Don't Know  (.) Missing |
|  | INDFMPIR (2011-2020) | The ratio of family income to poverty | The ratio of family income to poverty guidelines | Continuous  (.) Missing |
|  | HIQ011  (2011-2020) | Covered by health insurance | {Are you/Is SP} covered by health insurance or some other kind of health care plan? [Include health insurance obtained through employment or purchased directly as well as government programs like Medicare and Medicaid that provide medical care or help pay medical bills.] | 1. Yes  2. No  7. Refused  9. Don’t know  (.) Missing |
|  | HIQ210  (2011-2020) | Time when no insurance in past year | In the past 12 months, was there any time when {you/SP} did not have any health insurance coverage? | 1. Yes  2. No  7. Refused  9. Don’t know  (.) Missing |
|  | **Behavioral factors** | | | |
|  | SMQ020 (2011-2020) | Smoked at least 100 cigarettes in life | {Have you/Has SP} smoked at least 100 cigarettes in {your/his/her} entire life? | 1. Yes  2. No  3. Refused  4. Don’t know  (.) Missing |
|  | PAD680 (2011-2020) | Minutes sedentary activity | How much time {do you/does SP} usually spend sitting on a typical day? | Continuous  (.) Missing |
|  | PAQ605 (2011-2020) | Vigorous work activity | Does {your/SP's} work involve vigorous-intensity activity that causes large increases in breathing or heart rate like carrying or lifting heavy loads, digging or construction work for at least 10 minutes continuously? | 1. Yes  2. No  7. Refused  9. Don’t know  (.) Missing |
|  | PAQ620 (2011-2020) | Moderate work activity | Does {your/SP's} work involve moderate-intensity activity that causes small increases in breathing or heart rate such as brisk walking or carrying light loads for at least 10 minutes continuously? |  |
|  | PAQ635 (2011-2020) | Walk or bicycle | In a typical week {do you/does SP} walk or use a bicycle for at least 10 minutes continuously to get to and from places? |  |
|  | PAQ650 (2011-2020) | Vigorous recreational activities | In a typical week {do you/does SP} do any vigorous-intensity sports, fitness, or recreational activities that cause large increases in breathing or heart rate like running or basketball for at least 10 minutes continuously? |  |
|  | PAQ665 (2011-2020) | Moderate recreational activities | In a typical week {do you/does SP} do any moderate-intensity sports, fitness, or recreational activities that cause a small increase in breathing or heart rate such as brisk walking, bicycling, swimming, or volleyball for at least 10 minutes continuously? |  |
|  | **Clinical factors** | | | |
|  | MCQ160A (2011-2020) | Doctor ever said you had arthritis | Has a doctor or other health professional ever told {you/SP} that {you/s/he} . . .had arthritis? | 1. Yes  2. No  7. Refused  9. Don’t know  (.) Missing |
|  | KIQ022  (2011-2020) | Ever told you had weak/failing kidneys? | {Have you/Has SP} ever been told by a doctor or other health professional that {you/s/he} had weak or failing kidneys? Do not include kidney stones, bladder infections, or incontinence. |  |
|  | MCQ010 (2011-2020) | Ever been told you have asthma | Has a doctor or other health professional ever told {you/SP} that {you have/s/he/SP has} asthma? |  |
|  | MCQ160L (2011-2020) | Ever told you had any liver condition | Has a doctor or other health professional ever told {you/SP} that {you/s/he} . . .had any kind of liver condition? |  |
|  | MCQ220 (2011-2020) | Ever told you had cancer or malignancy | {Have you/Has SP} ever been told by a doctor or other health professional that {you/s/he} had cancer or a malignancy of any kind? |  |
|  | MCQ160B (2011-2020) | Ever told had congestive heart failure | Has a doctor or other health professional ever told {you/SP} that {you/s/he} . . .had congestive heart failure? |  |
|  | MCQ160C (2011-2020) | Ever told you had coronary heart disease | Has a doctor or other health professional ever told {you/SP} that {you/s/he} . . .had coronary heart disease? |  |
|  | MCQ160D (2011-2020) | Ever told you had angina/angina pectoris | Has a doctor or other health professional ever told {you/SP} that {you/s/he} . . .had angina, also called angina pectoris? |  |
|  | MCQ160E (2011-2020) | Ever told you had heart attack | Has a doctor or other health professional ever told {you/SP} that {you/s/he} . . .had a heart attack (also called myocardial infarction)? |  |
|  | MCQ160F (2011-2020) | Ever told you had a stroke | Has a doctor or other health professional ever told {you/SP} that {you/s/he} . . .had a stroke? |  |
|  | SLQ050  (2011-2020) | Ever told doctor had trouble sleeping | {Have you/Has SP} ever told a doctor or other health professional that {you have/s/he has} trouble sleeping? |  |
|  | **Anthropometric and biomarkers** | | | |
|  | LBDNENO (2011-2020) | Neutrophils | Segmented neutrophils number (1000c cells/uL) | Continuous  (.) Missing |
|  | LBXWBCSI (2011-2020) | White blood cell count | White blood cell count (1000 cells/uL) |  |
|  | LBXRDW (2011-2020) | Red cell distribution width | Red cell distribution width (%) |  |
|  | LBXMCVSI (2011-2020) | Mean cell volume | Mean cell volume (fL) |  |
|  | LBXPLTSI  (2011-2020) | Platelet count | Platelet count (1000 cells/uL) |  |
|  | LBXSGTSI (2011-2020) | Gamma Glutamyl Transferase | Gamma glutamyl transferase (U/L) |  |
|  | LBXSASSI  (2011-2020) | Alanine aminotransferase | Alanine aminotransferase (U/L) |  |
|  | LBXSAPSI  (2011-2020) | Alkaline phosphatase | Alkaline phosphatase (U/L) |  |
|  | LBDEONO (2011-2020) | Eosinophils | Eosinophils number (1000 cells/uL) |  |
|  | LBDBANO (2011-2020) | Basophils | Basophils number (1000 cells/uL) |  |
|  | LBXGH  (2011-2020) | Glycohemoglobin | Glycohemoglobin (%) |  |
|  | LBDSTRSI (2011-2020) | Triglyceride | Triglyceride (mmol/L) |  |
|  | LBDTCSI (2011-2020) | Total cholesterol | Total cholesterol (mmol/L) |  |
|  | BMXBMI (2011-2020) | Body mass index | Body mass index (kg/m²) |  |
|  | LBDHDDSI (2011-2020) | Direct high-density lipoprotein cholesterol | Direct high-density lipoprotein cholesterol (mmol/L) |  |
|  | LBXSNASI (2011-2020) | Sodium | Sodium (mmol/L) |  |
|  | LBDSTBSI (2011-2020) | Total bilirubin | Total bilirubin (umol/L) |  |
|  | LBXHGB (2011-2020) | Hemoglobin | Hemoglobin (g/dL) |  |
|  | LBXHCT (2011-2020) | Hematocrit | Hematocrit (%) |  |
|  | URXUMS (2011-2020) | Albumin | Albumin, urine (mg/L) |  |
|  | LBDSALSI  (2011-2020) |  | Albumin, refrigerated serum (g/L) |  |
|  | LBDMONO (2011-2020) | Monocyte number | Monocyte number (1000 cells/uL) |  |
|  | LBDLYMNO (2011-2020) | Lymphocyte number | Lymphocyte number (1000 cells/uL) |  |
|  | LBXSKSI (2011-2020) | Potassium | Potassium (mmol/L) |  |
|  | LBDSUASI  (2011-2020) | Uric acid | Uric acid (umol/L) |  |
|  | URXCRS (2011-2020) | Creatinine | Creatinine, urine (umol/L) |  |
