# Supplementary material for: Machine learning-based predictive modeling of depression in hypertensive populations
Source: PLoS One. 2022 Jul 29;17(7):e0272330. doi: 10.1371/journal.pone.0272330 (PMC9337649; doi:10.1371/journal.pone.0272330)
Supplement: S1 Table — (DOCX) [file pone.0272330.s002.docx]

# **S1 Table. The 2 X 2 contingency table.**

|  |  | **Actual values** | |
| --- | --- | --- | --- |
|  |  | **Positive (1)** | **Negative (0)** |
| **Predicted values** | **Positive (1)** | TP | FP |
|  | **Negative (0)** | FN | TN |

The columns in confusion matrix represent the actual values of outcome variable from the test data and the rows represent the predicted values obtained from the machine learning model on test data.

- TP: True Positives which tells that the observations which are positive in test data and are also predicted positive by the machine learning model.
- TN: True Negatives which tells that the observations which are negative in test data and are also predicted negative by the machine learning model.
- FP: False Positives which tells that the observations which are positive in test data and are wrongly predicted as negative by the machine learning model.
- FN: False Negatives which tells that the observations which are negative in test data and are wrongly predicted as positive by the machine learning model

Based on TP, TN, FP and FN, various measures of diagnostic performance are calculated.

- Sensitivity = TP / (TP + FN)
  - Sensitivity determines the proportion of actual positives that are correctly identified.
- Specificity = TN / (TN + FN)
  - Specificity determines the proportion of actual negatives that are correctly identified.
- Accuracy = [(TP + TN) / (TP + TN + FP + FN)]
  - Accuracy is a ratio of total correctly classified observations divided by total number of observations.
- Precision = TP / (TP + FP)
  - Precision is the ratio of number of TP observations and total TP observations.
- Recall = TP / (TP + FN)
  - Recall is defined as the ratio of the total number of correctly classified positive classes and the total number of positive classes.
- F1-score = 2 * (Precision * Recall) / (Precision + Recall)
  - F1-score is the harmonic mean of precision and recall. If F1-score is high, then both precision and recall is better.
- Desired values of all measures for the classification model are near to 1.
