# Supplementary material for: Machine learning-based predictive modeling of depression in hypertensive populations
Source: PLoS One. 2022 Jul 29;17(7):e0272330. doi: 10.1371/journal.pone.0272330 (PMC9337649; doi:10.1371/journal.pone.0272330)
Supplement: S2 Table — (DOCX) [file pone.0272330.s003.docx]

# **S2 Table. The list of variables selected from the Boruta and LASSO algorithms and stepwise backward elimination.**

| **Feature selection** | **Variable** |
| --- | --- |
| **Boruta algorithm** | Confirmed: Target^a^ ~ RIDAGEYR + RIDRETH3 + DMDMARTZ + DMDEDUC2 + RIAGENDR + INDFMPIR + URXUMS + LBXSATSI + LBXSASSI + LBDSALSI + URXCRS + LBDTCSI + LBXSGTSI + LBDNENO + LBXHGB + LBXGH + LBDHDDSI + LBXHCT + LBDLYMNO + LBDMONO + LBDSTRSI + LBDSUASI + LBXWBCSI + LBXPLTSI + LBXRDW + LBXMCVSI + BMXBMI + MCQ010 + KIQ022 + MCQ160A + MCQ160L + MCQ160M + SMQ020 + HIQ011 + PAD680 + PAQ665 + SLQ050 + MCQ^b^  Tentative: HIQ210 + LBDSTBSI + LBXSAPSI + MCQ220  Final: Target^a^ ~ RIDAGEYR + RIDRETH3 + DMDMARTZ + DMDEDUC2 + RIAGENDR + INDFMPIR + URXUMS + LBXSATSI + LBXSASSI + LBDSALSI + URXCRS + LBDTCSI + LBXSGTSI + LBDNENO + LBXHGB + LBXGH + LBDHDDSI + LBXHCT + LBDLYMNO + LBDMONO + LBDSTRSI + LBDSUASI + LBXWBCSI + LBXPLTSI + LBXRDW + LBXMCVSI + BMXBMI + MCQ010 + KIQ022 + MCQ160A + MCQ160L + MCQ160M + MCQ220 + SMQ020 + HIQ011 + HIQ210 + PAD680 + PAQ665 + SLQ050 + MCQ^b^ |
| **LASSO algorithm** | Final: Target^a^~ RIDAGEYR + RIDRETH3 + DMDMARTZ + DMDEDUC2 + RIAGENDR + INDFMPIR + LBXSAPSI + URXUMS + LBXSASSI + LBDSALSI + URXCRS + LBDTCSI + LBDEONO + LBXSGTSI + LBDNENO + LBXHGB + LBXGH + LBXSKSI + LBDLYMNO + LBXSNASI + LBDSTRSI + LBDSUASI + LBXPLTSI + BMXBMI + MCQ010 + KIQ022 + MCQ160A + MCQ160L + MCQ160M + MCQ220 + SMQ020 + HIQ011 + HIQ210 + PAD680 + PAQ605 + PAQ620 + PAQ635 + PAQ650 + PAQ665 + SLQ050 + MCQ^b^ |
| **Stepwise backward elimination** | Final: Target^a^ ~ RIDAGEYR + RIDRETH3 + DMDMARTZ + DMDEDUC2 + RIAGENDR + INDFMPIR + LBXSASSI + URXCRS + LBDTCSI + LBXHGB + LBXGH + LBDLYMNO + LBXSNASI + LBDSTRSI + LBDSUASI + LBXWBCSI + LBXPLTSI + MCQ010 + KIQ022 + MCQ160A + MCQ160L + MCQ220 + SMQ020 + HIQ210 +PAD680 + PAQ605 + PAQ650 + PAQ665 + SLQ050 + MCQ^b^ |

^a^Target is a target variable for predictive modeling (i.e., depression) and this was created by combining existing variables (DPQ010-090) in the dataset; please see the codebook below for further details.

^b^MCQ is a new variable that was created by combining existing variables (MCQ160b, MCQ160c, MCQ160d, MCQ160e, MCQ160f); please see the codebook below for further details.
