# Supplementary material for: Machine learning-based predictive modeling of depression in hypertensive populations
Source: PLoS One. 2022 Jul 29;17(7):e0272330. doi: 10.1371/journal.pone.0272330 (PMC9337649; doi:10.1371/journal.pone.0272330)
Supplement: S3 Table — (DOCX) [file pone.0272330.s004.docx]

# **S3 Table. Average metrics of six models trained with Boruta and LASSO algorithms.**

| **Boruta** | | | | | | |
| --- | --- | --- | --- | --- | --- | --- |
| **Model** | **AUC** | **Accuracy** | **Precision** | **Sensitivity** | **Specificity** | **F1-score** |
| **ANN** | **0.803** | 0.698 | 0.958 | 0.692 | **0.771** | 0.803 |
| **Random forest** | 0.771 | 0.685 | 0.948 | 0.675 | 0.759 | 0.790 |
| **AdaBoost** | 0.760 | 0.670 | 0.952 | 0.660 | 0.749 | 0.779 |
| **Stochastic gradient boosting** | 0.799 | 0.700 | 0.949 | 0.699 | 0.743 | 0.801 |
| **XGBoost** | 0.801 | 0.706 | 0.956 | 0.683 | 0.762 | 0.800 |
| **SVM** | 0.759 | **0.761** | **0.966** | **0.729** | 0.721 | **0.843** |
| **LASSO** | | | | | | |
| **ANN** | **0.811** | 0.703 | 0.955 | 0.646 | 0.760 | 0.800 |
| **Random forest** | 0.770 | 0.684 | 0.953 | 0.670 | **0.763** | 0.789 |
| **AdaBoost** | 0.760 | 0.669 | 0.946 | 0.658 | 0.733 | 0.778 |
| **Stochastic gradient boosting** | 0.800 | 0.700 | 0.950 | 0.699 | 0.739 | 0.808 |
| **XGBoost** | 0.803 | 0.690 | 0.954 | 0.681 | 0.760 | 0.789 |
| **SVM** | 0.763 | **0.769** | **0.960** | **0.765** | 0.729 | **0.851** |

The highest value was bolded.

AUC: area under the receiver operating characteristic curve; ANN: artificial neural network; SVM: support vector machine
